# Kameela—The New Anthelmintic

**Published:** 1859-05

**Authors:** J. M. Boisnot

**Affiliations:** Philadelphia


*Kameela—The New Anthelmintic.*—By J. M. Boisnot, M.D., of Philadelphia.

"Kameela is the reddish-brown powder which clothes the capsules of the Rottlera Tinctoria, one of the euphorbiacæ. This middle sized, erect, branching tree, named after the Rev. Dr. Rottler, who resided at Tranquebar for many years, in the character of a Danish Missionary, is found in the hilly parts of India, along the base of the Himalayas, from Assam to near Peshawur, in central India, at the northern Circurs, in Mysore, and at Parrell Hill, near Bombay; besides in Ceylon, China, Australia and Arabia. The capsules of the fruit of this tree, which are about the size of a small cherry, are clothed with abundance of deep red granular powder, easily rubbed off, much esteemed as a dye for silk. When the capsules are ripe, in February or March, they are gathered, and the powder carefully brushed off Dr. Anderson, Professor of Chemistry in Glasgow University, who has written on the coloring matter of the Rottlera tinctoria, found it to possess a substance which he named Rottlerine. It consists of yellew crystals, having the form of minute plates, and a fine satiny lustre; it is insoluble in water, and sparingly soluble in cold alcohol. Kameela, Kameyla, Kamila, Kamala, are the different names by which writers have designated this brick-red colored powder. Mr. Hanbury states that Kameela resembles lycopodium in the difficulty with which it is mixed with water, and in the manner in which it ignites when thrown into the air over the flame of a candle. Dr. Anderson, in the Indian Annals of Medicine for October, 1855, was the first to bring this medicine before the profession. In 95 cases of tape worm treated with Kameela by him, in two only was no worm expelled. With Kameela there is no unpleasant effect. It is not even necessary to take a dose of purging medicine as a preparative; and beyond a trifling amount of nausea and griping in some instances, no unpleasant effects are experienced; while by far the greater number of persons to whom it is administered suffer no inconvenience, beyond what they would from a dose of ordinary purging medicine. The certainty of its results are thus spoken of by Dr. Gordon: "I have never seen the remedy fail in removing the worm, in a case where there were unequivocal symptoms of its presence."—*Braithwaite's Retrospect*, part xxxv, p. 263, and part xxxviii, p. 88.

The want of an anthelmintic that could be relied on in the treatment of tape-worm, has long been felt by the public and the profession, but if we can judge by the success which has been attendant on the use of Kameela, in the hands of a few physicians, this want has been supplied, and the uncertainty of the remedies formerly used supplanted by the certainty of this.

That the existence and benefits of so valuable a remedy as this might be more thoroughly known to the profession, I have thought it not altogether inappropriate to present it to the numerous readers of the Reporter, even though my experience with it is limited to a single case.

Mrs. K., æt. 40, came under my care with the usual symptoms of tape worm, from which she had been suffering for the last five years; her health, previous to that time, having been, as she expressed it, perfect. After a dose of purgative medicine, Kameela was given in doses of grs. x. every 4 hours; slight nausea followed each dose, but no purgation until after the fifth dose had been taken; after six doses, or ʒj of the powder had been taken, a tape-worm, measuring 14 feet, came away. Since that time she has been steadily improving, with absence of all those distressing sensations caused by the presence of the worm in the bowels. In addition to the pieces passed per rectum, the most indicative symptoms were distressing pain in the epigastric region, with feeling of impending suffocation, and great depression of spirits and irritability of temper. The tenesmus and purging was excessive until the worm came away, and was attributed to the action of the medicine upon it, from the fact that ninety-two grains of the powder were given in twenty-three grain doses every half hour, a few days after its discharge, with no unpleasant feeling beside slight nausea and weight, and even requiring a dose of purgative medicine to move the bowels.

The facility with which Kameela may be taken, the mildness of its operation, and, above all, the certainty of its results, are the principal features to recommend it, while its scarcity and high price in a slight degree oppose its use, but with the hope soon to see the former established, and the latter removed, we recommend it to the profession.—*Med. & Sur. Reporter.*

